# Dramatic Reduction of Distant Pancreatic Metastases Using Local Light Activation of Verteporfin with Nab-Paclitaxel

**DOI:** 10.3390/cancers13225781

**Published:** 2021-11-18

**Authors:** Michael Pigula, Zhiming Mai, Sriram Anbil, Myung-Gyu Choi, Kenneth Wang, Edward Maytin, Brian Pogue, Tayyaba Hasan

**Affiliations:** 1 Department of Chemistry, Scripps Research, La Jolla, CA 92037, USA; mpigula@scripps.edu; 2 Wellman Center for Photomedicine, Massachusetts General Hospital and Harvard Medical School, Boston, MA 02114, USA; Mai.Zhiming@mgh.harvard.edu; 3 David Geffen School of Medicine at UCLA, Los Angeles, CA 90095, USA; Sanbil@mednet.UCLA.edu; 4 Catholic Research Institute of Medical Science, The Catholic University of Korea, Seoul 137-040, Korea; choim@catholic.ac.kr; 5 Division of Gastroenterology and Hepatology, Mayo Clinic, Rochester, MN 55902, USA; wang.kenneth@mayo.edu; 6 Department of Dermatology, Cleveland Clinic, Cleveland, OH 44195, USA; maytine@ccf.org; 7 Department of Engineering Sciences, Dartmouth College, Hanover, NH 03755, USA; Brian.W.Pogue@dartmouth.edu

**Keywords:** photodynamic therapy, pancreatic cancer, metastatic disease, abscopal effect

## Abstract

**Simple Summary:**

Surgical resection is currently the only potentially curative option for patients with pancreatic adenocarcinoma (PDAC). However, 80% of patients are ineligible for surgery due to the presence of invasive disease or distant metastases at the time of diagnosis. Treatment strategies geared towards reclassifying these patients as surgical candidates by reducing metastatic burden represents a promising approach to improving long-term survival. We investiaged the efficacy of a photodynamic therapy (PDT) based treatment regimen at preventing the spread and reducing the burden of metastases in animal models of PDAC. We demonstrate that PDT in combination with the first-line chemotherapeutic nab-paclitaxel dramatically inhibits (up to 100%) the eventual development of metastases in models of early stage PDAC, and completely eliminates metastasis in 55% of animals with already established distant disease in late-stage models. Our findings suggest that PDT-based treatment regimens may enable reclassification of patients with previously inoperable disease as surgical candidates.

**Abstract:**

Despite substantial drug development efforts, pancreatic adenocarcinoma (PDAC) remains a difficult disease to treat, and surgical resection is the only potentially curative option. Unfortunately, 80% of patients are ineligible for surgery due to the presence of invasive disease and/or distant metastases at the time of diagnosis. Treatment strategies geared towards reclassifying these patients as surgical candidates by reducing metastatic burden represents the most promising approach to improve long-term survival. We describe a photodynamic therapy (PDT) based approach that, in combination with the first-line chemotherapeutic nab-paclitaxel, effectively addresses distant metastases in three separate orthotopic PDAC models in immunodeficient mice. In addition to effectively controlling local tumor growth, PDT plus nab-paclitaxel primes the tumor to elicit systemic effects and reduce or abrogate metastases. This combination dramatically inhibits (up to 100%) the eventual development of metastases in models of early stage PDAC, and completely eliminates metastasis in 55% of animals with already established distant disease in late-stage models. Our findings suggest that this light activation process initiates local biological and/or physiological changes within the tumor microenvironment that can be leveraged to treat both localized and distant disease, and potentially reclassify patients with previously inoperable disease as surgical candidates.

## 1. Introduction

Pancreatic ductal adenocarcinoma (PDAC) continues to be a devastating disease, with a 5-year survival rate of 10% [1]. Surgical resection combined with chemo and/or radiotherapy is currently the only potentially curative treatment. However, upwards of 90% of patients are diagnosed with advanced disease and committed to chemo- and/or radiotherapy regimens that only marginally extend overall survival [2]. Despite significant advances in our understanding of PDAC biology, progression free survival (PFS) and overall survival currently stand at 9 and 19 months, respectively, for patients with borderline or locally advanced PDAC [3]. PFS and overall survival for those with confirmed metastatic disease drops to 6 and 11 months [4]. Clearly, more effective therapeutic approaches that increase the proportion of patients eligible for surgery are desperately needed.

Here, we present a verteporfin–photodynamic therapy (PDT) based approach that, when combined with nab-paclitaxel (NabP), effectively inhibits metastatic colonization and eliminates established distant metastases in early- and late-stage models of PDAC, respectively. Our findings suggest a neoadjuvant approach by which select patients with metastatic disease may qualify for surgical resection. In addition, this approach may prevent metastatic colonization in patients with earlier-stage disease. Given that the presence of confirmed metastases currently precludes surgical intervention, the anti-metastatic effects of combined PDT and NabP may offer a potentially curative surgical opportunity for this patient population.

Unfortunately, due to the lack of screening technologies and asymptomatic nature of early-stage PDAC, approximately 60% of patients present with disease that has already metastasized [5]. Only 10% of patients are eligible for resection at the time of diagnosis, with a small proportion diagnosed with borderline or locally advanced PDAC, typically defined as <180° abutment of the superior mesenteric artery (SMA) and >180° encasement of the SMA, respectively [1,6]. Significant progress has been made in the past decade for patients with resectable or borderline resectable disease, with disease-free survival approaching 2 years following adjuvant or neoadjuvant chemotherapy regimens, such as FOLFIRINOX (fluorouracil, leucovorin, irinotecan, and oxaliplatin) and nab-paclitaxel plus gemcitabine [2]. Additionally, a recent phase II study suggests that nab-paclitaxel plus gemcitabine may facilitate the downstaging of patients with nonresectable locally advanced disease and convert a significant proportion into candidates for surgery [7]. Although only two-thirds of patients with borderline resectable disease will go on to receive surgery, their R0 resection rates (90%) and overall survival (~30 months) are similar to those with initially resectable disease [3], suggesting that surgery represents the best chance of long-term survival for these patients. Outcomes for those with locally advanced or metastatic disease are significantly worse, largely because surgery is rarely an option. Our study suggests that combined PDT and NabP may enable successful treatment and elimination of distant metastases, thereby downstaging patients with advanced disease to enable resection and better outcomes.

Photodynamic therapy utilizes the photochemical properties of small molecule photosensitizers to convert energy from visible or infrared light into locally cytotoxic reactive molecular species. Extensive work by our group and others has consistently demonstrated that PDT can increase intratumoral concentrations of chemotherapy [8,9] and sensitize refractory disease to traditional anticancer drugs [10]. In the context of PDAC, PDT has been shown to inhibit the formation of stem-like cell populations following treatment [9], as well as biochemically modulate the tumor microenvironment to improve the efficacy of chemotherapy [11]. PDT is FDA approved for a number of cancers, including skin, head and neck, and prostate, and was well tolerated and effective in two recent phase I-II trials for treatment of advanced PDAC [12,13]. A phase II trial of PDT + chemotherapy for patients with advanced or locally advanced PDAC (including those with limited metastases) is currently underway (VERTPAC02-NCT03033225) [14].

Although PDT is delivered locally to primary tumors, it is known to induce a strong systemic response, and preclinical studies of PDT vaccines and tumor rechallenge models have consistently demonstrated the formation of an anti-tumor memory following treatment [15]. PDT-based combination regimens have been shown to control the growth of distant established tumors in animal models [16] and inhibit the formation of metastases [17]. However, given the different models and timing of treatment used in these studies, it is often unclear whether the systemic effects of PDT reduce the metastatic potential of primary tumors, inhibit metastatic colonization, or represent true abscopal effects, whereby already established metastatic colonies are cytoreduced following local treatment of the primary tumor.

In this study, we aim to separately investigate these possibilities using three mouse models of pancreatic cancer, each with a well-defined metastatic status at the time of treatment. We demonstrate that PDT in combination with nab-paclitaxel and gemcitabine (Gem) effectively inhibits the formation of metastases in an early-stage model of PDAC, and clears established distant metastatic colonies in a substantial fraction of animals in a late-stage model. Given its mild and well-characterized side effects and the desperate need for innovative therapies, this PDT-based treatment strategy represents a promising and rapidly translatable approach that may increase the proportion of patients eligible for surgical resection.

## 2. Materials and Methods

### 2.1. Cell Culture and Reagents

AsPC-1 (metastatic cells derived from ascites) and MIA PaCa-2 (primary tumor derived) human pancreatic cancer cells were purchased from ATCC (Manassas, VA, USA), cultured as per the manufacturer’s instructions, and confirmed negative for mycoplasma contamination. Visudyne (liposomal Verteporfin) photosensitizer was a kind gift from QLT Inc (Vancouver, BC, Canada). Nab-paclitaxel, comprising 90% human albumin and 10% active ingredient (paclitaxel), was obtained from Abraxis BioScience (Bridgewater, NJ, USA). Gemcitabine HCl was purchased from Sandoz Inc. (Princeton, NJ, USA).

### 2.2. Orthotopic Mouse Model

All animal study protocols were approved by the Institutional Animal Care and Use Committee at the Massachusetts General Hospital. Orthotopic pancreatic tumors were initiated in 4–6 week-old male Swiss nude mice (Cox Breeding Laboratories, Cambridge, MA, USA). The animal was anesthetized with ketamine/xylazine, and on its left side a 1 cm long incision was made laterally over the spleen. The pancreas was exposed by gently pulling the spleen out of the incision, and 1 × 10^6^ AsPC-1 or MIA PaCa-2 cells suspended in 50 µL of 50% Matrigel (BD Biosciences, San Diego, CA, USA) were injected into the body of the pancreas. The incision was closed with 4–0 monofilament suture. Animals were monitored over the course of the experiment per study protocols. At the study endpoint, animals were sacrificed, and tumor tissue excised to record weight. Liver, iliac lymph nodes, and lungs were harvested to evaluate metastatic burden by qRT-PCR.

### 2.3. In Vivo Treatment Regimen

Treatment efficacy was evaluated based on tumor weight. For experiments in which treatment was initiated on day 9 post implantation, tumors were approximately 30–50 mg (3–4 mm diameter), and for those initiated on day 24, tumors were approximately 300 mg (~8 mm diameter). All treatments were administered via tail vein injection. For groups receiving PDT, one hour following the injection of Visudyne (0.25 mg/kg), the pancreas was exteriorized as during implantation. The tumor was illuminated with 690 nm laser light (100 mW/cm2, 50 J/cm2). All mice treated with PDT received only a single dose of PDT. Following PDT, the incision was closed with 4–0 sutures. For animals receiving chemotherapy, each cycle consisted of nab-paclitaxel (25 mg paclitaxel/kg per cycle) and/or Gemcitabine (100 mg Gemcitabine/kg per cycle). Chemotherapy was administered starting day 9 (30 min post-PDT if appropriate), and again every 4 days for a total of 4 cycles. Untreated control animals received phosphate buffered saline at the appropriate time point.

### 2.4. Quantification of Metastatic Burden

A quantitative RT-PCR-based method (qRT-PCR) was used to detect human cancer cells in excised mouse samples of liver, iliac lymph nodes (containing surrounding muscle), and lungs. Tissue was immediately harvested from the sacrificed animal at the end of the experiment and snap frozen in liquid nitrogen. Frozen tissues were pulverized, and RNA was extracted using RNeasy Protect Mini Kits (Qiagen, CA, USA) according to the manufacturer’s instructions. Samples were reverse transcribed into cDNA, which served as PCR templates. Relative amount of human and mouse GAPDH genes were quantified using the cycle threshold obtained from qRT-PCR, and number of human cells per gram of tissue was calculated from a set of standard curves.

### 2.5. Statistical Analysis

All statistical analyses were carried out using appropriate tools in Graphpad Prism 8. Unless otherwise specified, tumor weights were plotted as mean with standard deviation error bars and analyzed by Kruskal–Wallis ANOVA with Dunn multiple comparisons. Metastases were plotted as medians and analyzed with Kruskal–Wallis ANOVA with Dunn multiple comparisons. Fisher’s exact test was used to analyze datasets comparing the presence or absence of metastases in mice following each treatment. Sample sizes are indicated in figure captions.

## 3. Results

### 3.1. PDT + Nab-Paclitaxel/Gemcitabine Completely Inhibits the Metastatic Potential of Early-Stage PDAC

**Figure 1:**
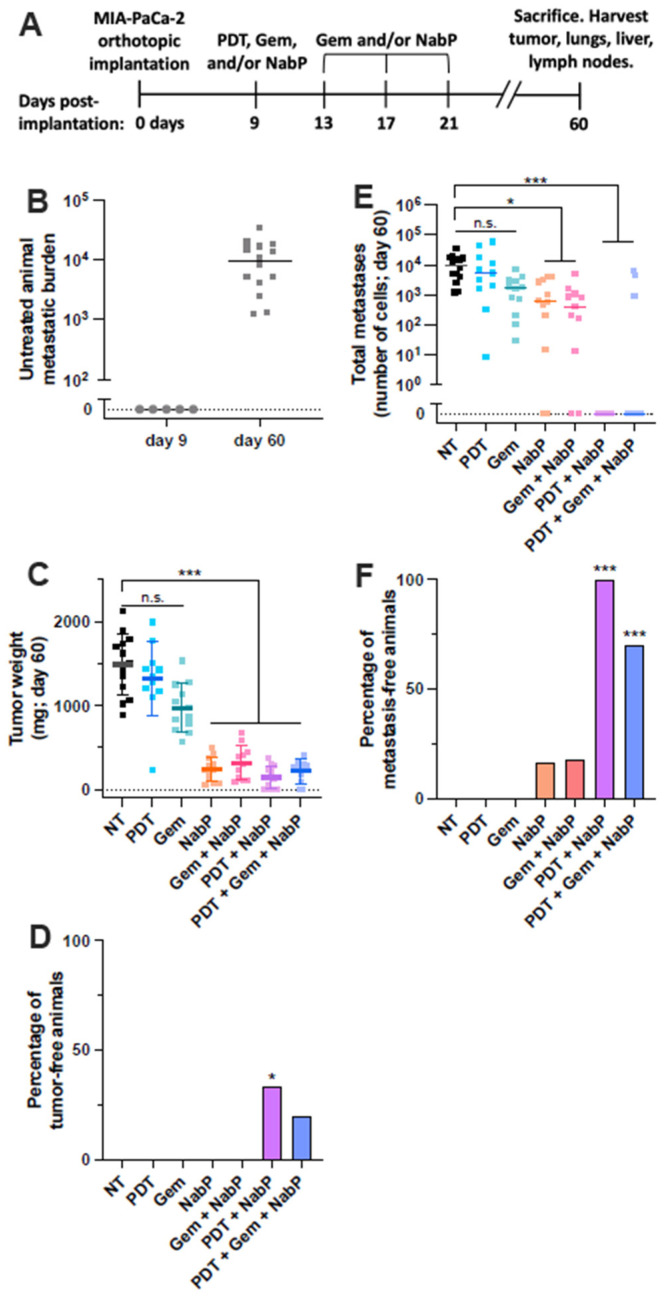
PDT + NabP completely inhibits metastatic colonization in a low-grade early stage PDAC model. (**A**) Premetastatic low-grade PDAC model experimental timeline. Treatment was initiated nine days following tumor implantations, and animals sacrificed and tissue analyzed after 60 days. (**B**) No untreated animals harbor metastases at the time of treatment nine days post-implantation, while 100% do by day 60. (**C**) NabP-based therapies exhibit similar degrees of primary tumor control. (**D**) PDT + NabP treatment results in a higher rate of primary tumor elimination than other treatment groups. (**E**,**F**) Mice in PDT + NabP-based treatment groups have significantly lower metastatic burden and lower rates of metastatic spread. *n* = 10–14 for all groups. N.s.: not statistically significant; * *p* < 0.05; *** *p* < 0.001.

The MIA PaCa-2 (MP2) cell line is derived from a primary pancreatic tumor, and generally exhibits less aggressive phenotypes compared to ascites-derived AsPC-1 cells, including increased sensitivity to gemcitabine [18] and a slower growth rate in vivo [19]. Here, a MP2 orthotopic model was used to simulate early stage, premetastatic disease to investigate the effect of PDT-based combination therapies on tumor metastasis rates (Figure 1A). On day 9 following implantation, no human cells were detected in the liver, lungs, or lymph nodes of five mice, confirming the absence of metastases at the time of treatment (Figure 1B). A sensitive qRT-PCR technique (see Methods section) was used to quantify human cell specific mRNA in each tissue, enabling detection of micrometastatic disease that non-invasive imaging techniques would not reveal. In contrast, all untreated mice contained metastatic disease by day 60. Mice bearing MP2 tumors were then treated with Gem, NabP, PDT, Gem + NabP, PDT + NabP, or PDT + Gem + NabP. Neither PDT nor Gem alone impacted primary tumor size 60 days post-implantation (Figure 1C). In contrast, all NabP containing regimens resulted in similar and significant primary tumor reduction, suggesting that NabP is the main driver in primary tumor response in this model. However, the addition of PDT to NabP did result in elimination of the primary tumors of 33% of mice (Figure 1D). Neither NabP nor Gem + NabP treatments resulted in complete elimination of the primary tumor. Similarly, all NabP containing treatments significantly reduced the overall metastatic burden 51 days following the initiation of treatment (Figure 1E). Furthermore, PDT + NabP completely inhibited the development of metastases in all mice, whereas less than 20% of NabP- or Gem + NabP-treated animals were metastasis-free at this time point (Figure 1F). Interestingly, the addition of Gem to the PDT + NabP regimen resulted in poorer, but statistically similar (*p* = 0.08), control of metastatic spread, as 30% of mice treated with this combination had detectable metastases. Similar results have been previously reported, wherein the addition of Gem to a NabP regimen did not result in any additional survival benefit in subcutaneous PDAC tumors [20]. This trend perhaps suggests that further study is needed before Gem is routinely used in this triple therapy, and for these reasons, Gem was excluded from the PDT + NabP regimens in subsequent experiments.

### 3.2. PDT + Nab-Paclitaxel Delays Metastatic Colonization in an Early-Stage Model of Aggressive PDAC

**Figure 2:**
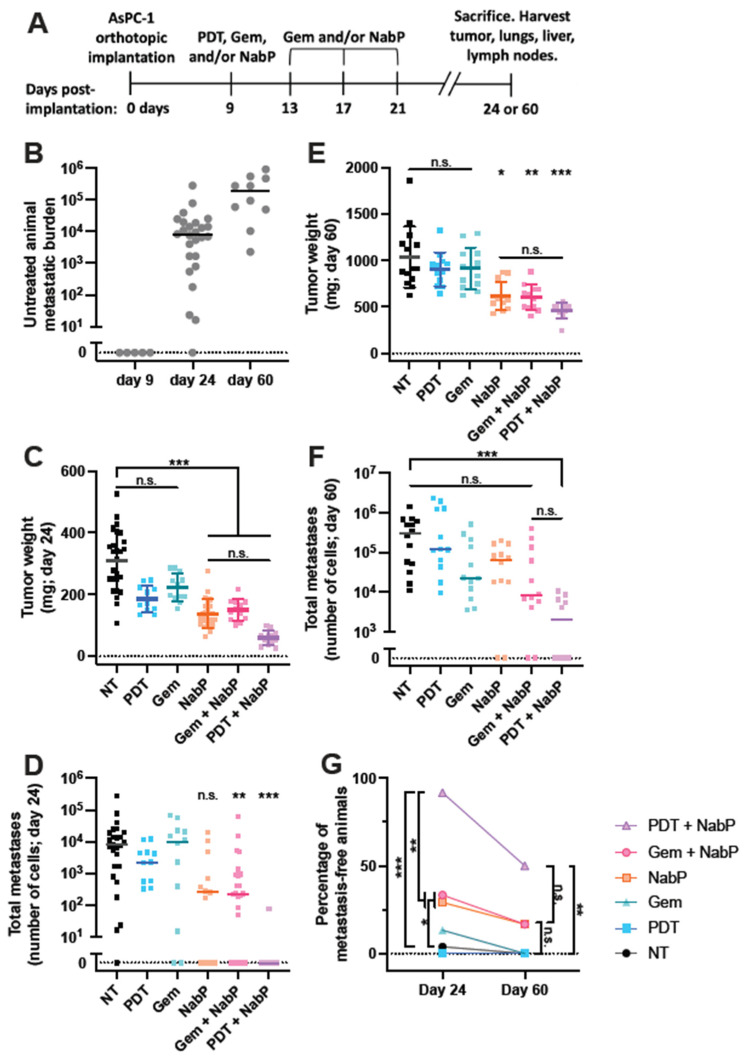
PDT + NabP delays the spread of metastases in an aggressive early-stage PDAC model. (**A**) Premetastatic aggressive PDAC model timeline. Treatment was initiated nine days following tumor implantations, and animals sacrificed and tissue analyzed after 24 or 60 days. (**B**) No metastases are detected at the time of treatment nine days following tumor implantation, while 96% and 100% of animals exhibit distant metastases 24 and 60 days post-implantation, respectively. (**C**,**D**) All NabP-containing regimens exhibit similar degrees of primary tumor control by day 24, though only Gem + NabP and PDT + NabP exhibit reduced metastatic burden at this time point. (**E**) All NabP-containing treatment groups exhibited similar reductions in primary tumor weight by day 60. (**F**) Only PDT + NabP-treated mice exhibited lower metastatic burden after 60 days. (**G**) NabP + PDT significantly inhibits the establishment of metastases in both short (24 days) and medium term (60 days) models. *n* = 10–26 for all groups. N.s.: not statistically significant; * *p* < 0.05; ** *p* < 0.01; *** *p* < 0.001.

Given its ability to completely inhibit the metastatic potential of PDAC in our model of early-stage disease, we aimed to evaluate whether PDT + NabP had similar efficacy in more aggressive models of PDAC. Mice bearing AsPC-1 tumors were treated with either PDT, Gem, NabP, Gem + NabP, or PDT + NabP, on day 9 post-implantation (Figure 2A), at which point animals do not harbor detectable metastases (Figure 2B). By days 24 and 60 post-implantation, 96% and 100% of untreated animals had metastatic disease, respectively. These endpoints were chosen to characterize acute and medium-term response to treatment, and to extrapolate the temporal dynamics of metastatic colonization in response to treatment.

At the day 24 endpoint, PDT- and Gem-treated mice did not exhibit a significant reduction in primary tumor size compared to untreated animals, whereas all NabP-based treatments resulted in significant and statistically similar control of primary tumors (Figure 2C). Gem + NabP- and PDT + NabP-treated mice also had a lower median metastatic burden than untreated animals (Figure 2D). However, the PDT + NabP combination prevented the development of metastases in 92% mice at this early time point, while only 29% and 33% of NabP- and Gem + NabP-treated animals were metastasis free, respectively (Figure 2G). Similarly, all three NabP containing therapies exhibited comparable control of the primary tumor on day 60 post-implantation (Figure 2E). However, neither NabP- nor Gem + NabP-treated animals had a significant reduction in metastatic burden nor incidence of metastases (Figure 2F,G), while the PDT + NabP regimen was effective at blocking the development of metastases in 50% of animals (Figure 2G). Taken together, these data indicate that PDT + NabP delays metastatic colonization in an early-stage model of aggressive PDAC.

### 3.3. PDT + Nab-Paclitaxel Cytoreduces Established Metastases in an Aggressive Late-Stage Model of PDAC

**Figure 3:**
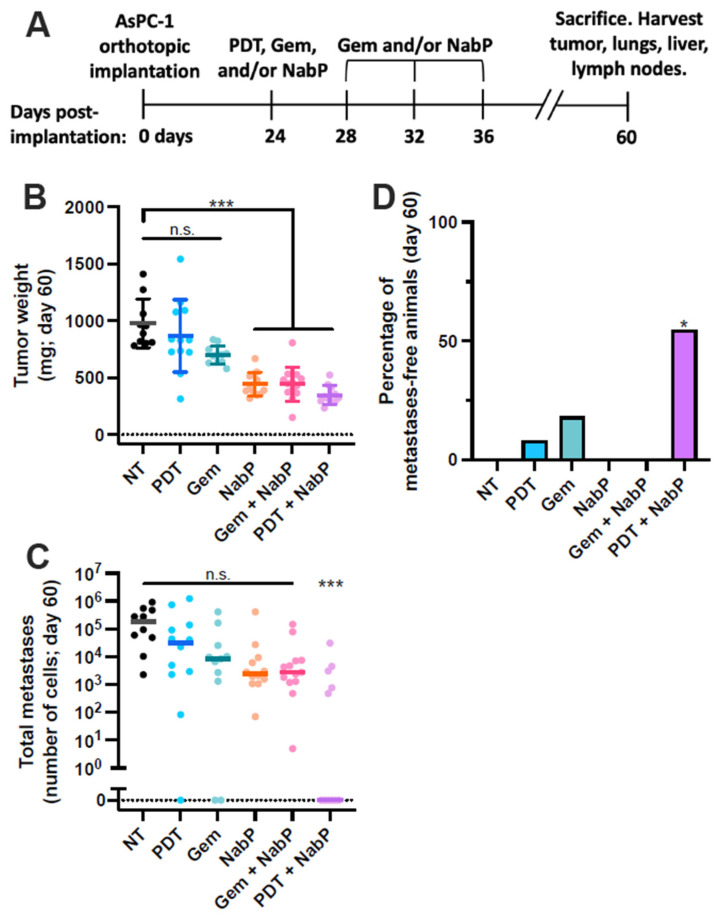
PDT + NabP eliminates distant established metastases. (**A**) Aggressive metastatic PDAC model timeline. Treatment was initiated 24 days following tumor implantations, and animals sacrificed and tissue analyzed after 60 days. (**B**) NabP-containing regimens exhibit similar degrees of primary tumor control. (**C**,**D**) PDT + NabP-treated mice exhibit significantly lower metastatic burden and incidence, and 55% of treated mice were metastasis free at the study end point. *n* = 10–13 for all groups. N.s.: not statistically significant; * *p* < 0.05; *** *p* < 0.001.

Given the efficacy of PDT + NabP in early-stage models of PDAC, we hypothesized that this combination may reduce metastatic burden in animals that already have established distant metastases. Since the majority of PDAC patients are diagnosed with stage IV disease, we simulated this scenario by initiating treatment on day 24 post-implantation of AsPC-1 cells (Figure 3A), when 96% of mice were known to have distant metastases based on the analysis shown in Figure 2B. Similar to treatment at earlier time points, the addition of PDT to NabP did not provide additional benefit in terms of primary tumor response when compared to Gem + NabP or NabP alone (Figure 3B). Strikingly, PDT + NabP led to a significant reduction in metastatic burden, and completely eliminated detectable distant metastases in 55% of mice (Figure 3C,D). In comparison, none of the other combinations tested in this study significantly reduced metastatic burden overall, and the PDT and Gem alone groups completed eliminated metastases in only 8% and 18% of animals, respectively (Figure 3C,D). Interestingly, animals treated with PDT + NabP exhibited a bimodal response, in that there was a population of mice that completely responded and harbored no distant metastases, and a separate population that had a metastatic burden comparable to chemotherapy alone treated mice (Figure 3C).

## 4. Discussion

The current study suggests that the addition of PDT to standard of care chemotherapy for PDAC may be an effective strategy to cytoreduce established metastases in patients with late stage PDAC well beyond what standard of care chemotherapy can, possibly enabling surgical resection. We demonstrate the utility of PDT in combination with NabP for robust control of PDAC in three well-characterized models of the disease across two cell lines. In mice treated prior to distant colonization of metastases, the addition of PDT to NabP did not improve primary tumor control over NabP alone in either short (24 days) or medium term (60 days) experiments. However, the proportion of animals that developed metastases after 60 days decreased from 83% to 0% in MIA PaCa-2 mice (Figure 1F), and 83% to 50% (*p* = 0.17) in AsPC-1 mice (Figure 2G) when PDT was added to the NabP regimen. Similarly, the incidence of metastases in mice bearing AsPC-1 tumors at day 24 was reduced from 71% to 8% upon the addition of PDT to NabP (Figure 2G). These data agree with prior reports that demonstrate AsPC-1 cells are highly chemoresistant both in vitro and in vivo [20,21], and have been shown to form more invasive and aggressive orthotopic tumors compared to other cell lines [22], in agreement with our own observations working with these models.

Consistent with the most common anatomical sites of metastases observed clinically [23], tissue from liver, lungs, and surrounding lymph nodes were individually analyzed for the presence of metastases at the end of each experiment (Figure S1). Liver metastases were observed in 100% of untreated animals harboring MiaPACA-2 tumors, and were consistently observed most frequently regardless of treatment (Figure S1A). Location of metastases was less defined in mice with AsPC-1 tumors, and which tended to lead to higher rates of lung and lymph node metastases (Figure S1B–D). The fact that AsPC-1 and MiaPACA-2 tumors preferentially metastasize to different organs likely reflects subtle differences in their biology. Interestingly, treatment regimen did not produce a consistent metastatic pattern across models, possibly due to idiosyncratic physiological response of individual mice, much as in individual patients.

Several prior studies have reported “abscopal” effects of PDT in reducing metastases in immunocompetent models of cancer [17,24]. Of particular interest is the fact that this study was performed in immunocompromised mice, specifically because we wanted to dissect any remote effect that the PDT process might have directly beyond the usual anticipated local shrinkage of tumors. We have put forward the concept of photodynamic priming (PDP) where the disturbance of the tumor microenvironment beyond the zone of necrosis leads to events that transiently impact local and remote sites [9,25,26]. This provides a window of opportunity for maximizing the effect of adjuvant treatments, such as chemotherapy and immune therapy. In the context of PDAC, our group has demonstrated that low-dose PDT followed by chemotherapy significantly reduces stem-like tumor cell populations that are known to contribute to treatment resistance and metastatic potential, in part through the downregulation of CD44 and CXCR4. This photodynamic priming strategy mitigates chemotherapeutic selection pressures typically imparted by high dose regimens and significantly inhibits primary tumor growth and metastatic burden in long-term experiments [9]. Additionally, PDT reduces the expression of CXCL12 and its receptor CXCR7 in a stroma-rich PDAC model [11], a paracrine-signaling axis that has been shown to facilitate metastatic colonization and disease progression [27]. Macrophage, dendritic cell, and neutrophil activation following PDT are all well documented [28]. Furthermore, cytokines have been shown to play an important, yet incompletely understood, role in the progression and metastatic colonization of many cancers and the associated immune response [29]. In the context of PDT, treatment of solid tumors has been shown to have significant effects on the peripheral immune cells and circulating cytokine levels [30,31].

We hypothesize that these mechanisms are at least partially responsible for the significant cytoreduction of distant disease in the T-cell deficient animal model described here. In fact, the immunocompromised mice likely underestimate both the local and systemic immune-mediated effects of PDT + NabP against this immune-privileged disease. These exciting findings point to the inherent ability of light activated neoadjuvant priming to alter the tumor microenvironment in ways that have dramatic effects on distant disease, possibly via the release of diffusible cytokines or activation of the innate immune system. Additional studies investigating these mechanisms in immunocompetent mouse models are warranted and may inform rationally designed PDT and immune checkpoint combination therapies. Furthermore, an in-depth characterization of similar metastatic models using cell lines with a range of chemotherapy/PDT sensitivities and metastatic potentials may lead to a deeper understanding of the impact of PDT and chemotherapy on metastasis control.

Although modern neoadjuvant chemotherapy regimens enable ~70% of borderline resectable patients to undergo resection [3], less than one-fifth of those with unresectable disease will be downstaged to the point of being surgical candidates [7]. Unfortunately, the majority of patients with PDAC present with stage IV disease characterized by the presence of distant metastatic colonies are by definition ineligible for resection. Approaches that increase the proportion of patients eligible for potentially curative surgical resection would represent a major advance for the treatment of PDAC, given the current dismal outcomes associated with the disease.

We demonstrate that PDT combined with NabP completely eliminates metastases in over 50% of animals that have established, distant disease at the time of treatment in an immunocompromised mouse model. Two early phase clinical trials investigating PDT for patients with inoperable, locally advanced PDAC suggest that PDT does indeed have the potential to help downstage disease to a point where it is amenable for resection. In one case, a patient received a relatively low dose (20J) of Visudyne-based PDT without follow up chemotherapy, and was subsequently determined to have resectable disease and underwent an R0 Whipple’s pancreaticoduodenectomy [12]. Similarly in another study, two patients receiving 100J of Photofrin-based PDT followed by Gem + NabP were later deemed to be candidates for distal pancreatomy [13]. In both cases, consistent with extensive clinical experience for other indications, PDT was well tolerated with minimal and manageable side effects (mild abdominal pain and temporary light sensitivity). PDT + NabP treatment was also found to be minimally toxic in AsPC-1- and MiaPACA-2-implanted mice (Figure S2). In both cases, mild weight loss of <10% was observed following the first round of treatment, which was resolved after approximately one week. Given the promising results from early phase clinical studies and those reported here, we believe that the addition of PDT to current standard chemotherapy regimens for those with advanced PDAC has the potential to be a rapidly translatable therapy for a patient population that currently has few therapeutic options.

While PDT + NabP results in uniform reduction in primary tumor size in the late-stage model, 45% of animals still have a substantial metastatic burden comparable to those treated with NabP only (Figure 3). This discrepancy suggests that subtle differences in the PDT procedure may have important biological outcomes, including immune activation. This could be in part due to the complexities of light penetration through heterogenous tumor tissue, which may be alleviated by more advanced light delivery and clinically used monitoring systems [32]. Furthermore, heterogeneity of chemotherapy-induced immunosuppression and differences in the tumor microenvironment between animals likely contribute to these variations in treatment response [33]. The bimodal distribution of metastatic burden following PDT + NabP, comprised of complete responders and non-responders, also suggests that there may be a population of animals (patients) that won’t benefit from this combination. Therefore, non-responding patient populations would likely benefit most from early detection technologies and screening strategies that are under investigation [34]. A more detailed analysis of the biological and biophysical mechanisms that dictate whether a particular animal responds to treatment, such as transcriptomic changes or immune profile of the tumor, may provide important insight as to the causes of treatment failure in this subpopulation and identify those that are most likely to respond favorably to treatment.

Pancreatic cancer continues to be a debilitating disease, in part due to the advanced stage of disease at which most patients are diagnosed. Unfortunately, approximately 60% of patients present with metastasized disease and are ineligible for potentially curative resection. Our findings indicate that by capturing the priming effect of PDT combined with front line chemotherapy, established distant metastases are eliminated in a significant fraction of animals, and this combination controls primary tumor growth for at least 7 weeks post treatment. Given recent promising reports of PDT in early-stage clinical trials and the limited treatment options available for these patients, we believe that our current exciting findings suggest that PDT has the potential to complement the current standard of care and provide this vulnerable patient population with an expanded set of therapeutic options. We anticipate that in immunocompetent subjects, the effect of PDP would be further enhanced by the additional benefit of immune activation, leading to a more robust response.

## Data Availability

All data is contained within the article.

## Supplementary Materials

The following are available online at https://www.mdpi.com/article/10.3390/cancers13225781/s1, Figure S1: Proportion of animals with detectable metastases by organ, Figure S2: Treatment toxicity.
