# Supplementary material for: Dramatic Reduction of Distant Pancreatic Metastases Using Local Light Activation of Verteporfin with Nab-Paclitaxel
Source: Cancers (Basel). 2021 Nov 18;13(22):5781. doi: 10.3390/cancers13225781 (PMC8616053; doi:10.3390/cancers13225781)
Supplement: Supplementary file 1 [file cancers-13-05781-s001.zip › supplementary updated.pdf]

Supplemental Figure Captions

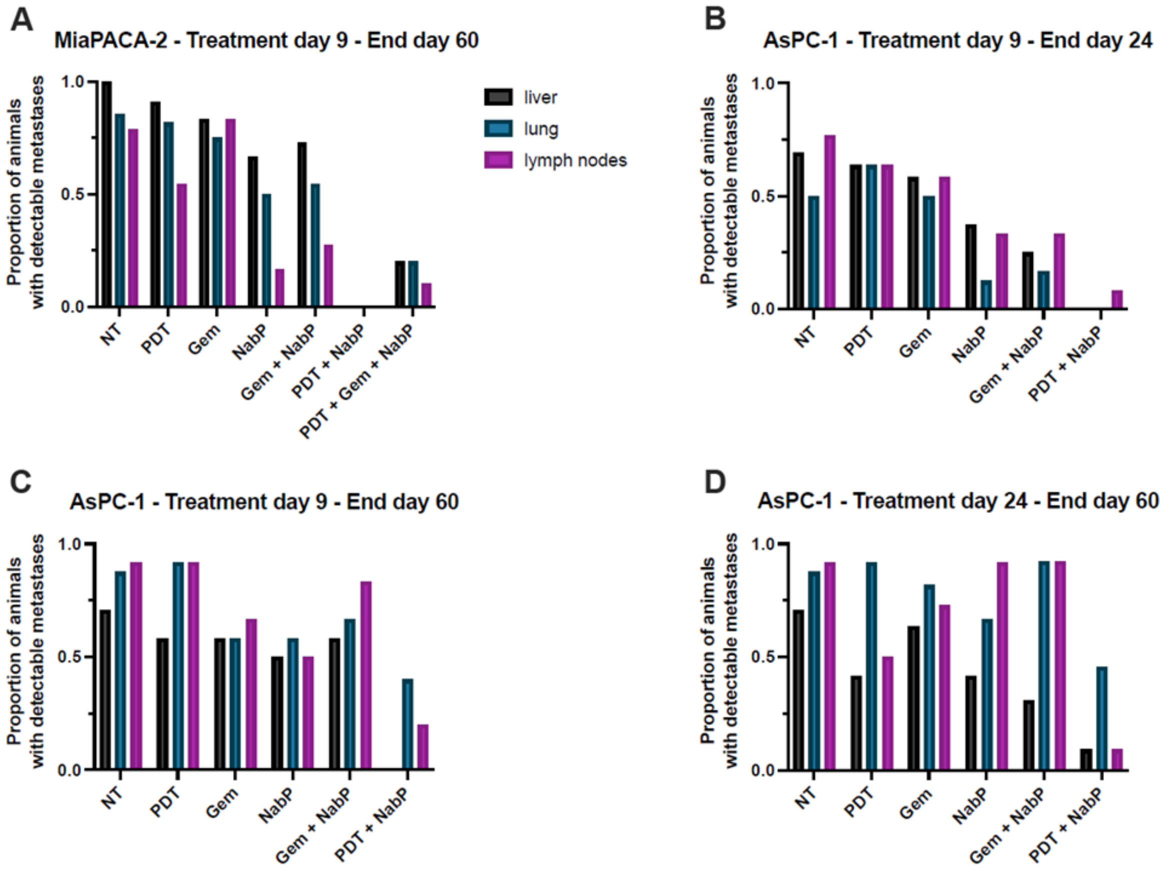

Figure S1. The proportion of animals with detectable metastases broken down by each organ (liver, lung, lymph nodes) in each experimental model. (**A**) MiaPACA-2 implanted animals were treated on day 9, which were sacrificed and each organ analyzed for the presence of metastases on day 60 post-implantation; AsPC-1 implanted animals treated on day 9 and sacrificed and analyzed for metastases on (**B**) day 24 post-implantation, or (**C**) day 60 post-implantation; (**D**) AsPC-1 implanted animals treated on day 24 and analyzed for metastases on day 60 post-implantation. The number of animals in each group is identical to those indicated in the main figures.

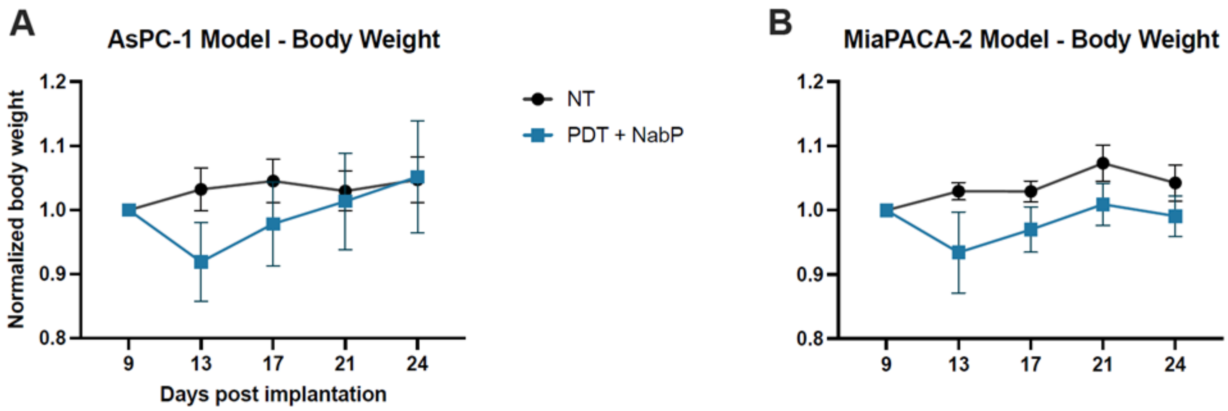

Figure S2. Normalized bodyweight curves following PDT + NabP treatment at day 9 for (**A**) AsPC-1 implanted mice (N = 12-14), and (**B**) MiaPACA-2 implanted mice (N = 8-11). Bodyweight of each individual animal at each time point was normalized to its weight on day 9, and are plotted as mean ± standard deviation. All IACUC protocols related to treatment toxicity and animal welfare were followed, including that which requires animals that experience weight loss of >20% be euthanized and removed from the study.
